# Genomics strategies for germplasm characterization and the development of climate resilient crops

**DOI:** 10.3389/fpls.2014.00068

**Published:** 2014-02-25

**Authors:** Robert J. Henry

**Affiliations:** Queensland Alliance for Agriculture and Food Innovation, University of QueenslandBrisbane, QLD, Australia

**Keywords:** genomics, evolution, climate adaptation, crops, wild crop relatives

## Abstract

Food security requires the development and deployment of crop varieties resilient to climate variation and change. The study of variations in the genome of wild plant populations can be used to guide crop improvement. Genome variation found in wild crop relatives may be directly relevant to the breeding of environmentally adapted and climate resilient crops. Analysis of the genomes of populations growing in contrasting environments will reveal the genes subject to natural selection in adaptation to climate variations. Whole genome sequencing of these populations should define the numbers and types of genes associated with climate adaptation. This strategy is facilitated by recent advances in sequencing technologies. Wild relatives of rice and barley have been used to assess these approaches. This strategy is most easily applied to species for which a high quality reference genome sequence is available and where populations of wild relatives can be found growing in diverse environments or across environmental gradients.

## NEED TO ADAPT CROPS TO NEW AND CHANGING ENVIRONMENTS AND THE ROLE OF GENOMICS

Agriculture needs significant increases in productivity to satisfy the expected growth in demand for food in the next few decades. The impact of climate variability and climate change on agricultural productivity is likely to be a major constraint to achieving increased food production. This makes the development of crop genotypes with resilience to climate change an important strategy for food security. Innovations in crop improvement based upon application of advanced genomics tools may be a way to address this need. The delivery of these technologies will require significant efforts in coordinated development and delivery of improved germplasm (Lybbert et al., 2013). Genomics allows resources available for crop adaptation to environmental stress to be characterized and utilized (Bansal et al., 2013). An evolutionary perspective may assist in the effective application of the power of genomic tools to the development of climate resilient crops adapted to a changing environment.

## GENOMIC ANALYSIS OF CROP EVOLUTION AND ADAPTATION TO CLIMATE CHANGE

Crop evolution has been relatively rapid under human selection over the last 10,000 years of agriculture. However, it is built on a very much longer period of evolution of wild crop relatives and the plant groups from which they are sourced. Understanding the processes and history of crop domestication and the evolution of related wild species provides critical knowledge to guide the development of crop varieties that are resilient to climate change in the future.

Analysis of wild plant populations provides evidence of factors contributing to success in periods of climate change. For example, hybridization between species may be an advantage in adapting to rapid climate change by providing new genetic combinations to cope with new environmental circumstances. Closely related species that can hybridize are more likely to survive than highly divergent species that cannot hybridize (Becker et al., 2013). Analysis of the genetics of populations growing across environmental gradients or from contrasting environments may be used to identify how plant populations adapt to climate under natural selection (Cronin et al., 2007). Sampling of populations at the same time over a long period of time can also be used to monitor adaptation to climate change but few sites have been sampled in the past in a way that allows this type of analysis to be conducted. Establishment of long term experiments of this type would be of great value. Recent dramatic improvements in genome analysis tools due to rapid advances in DNA sequencing technology make feasible research that should deliver much greater understanding of the relationships between wild and domesticated plant populations (Henry, 2012, 2013).

Recent fossil evidence suggests early diversification of groups of crop wild relatives such as the grasses (Prasad et al., 2011). The climate resilience of domesticated rice populations may be related to their evolutionary history. For example, expansion of the range of climates to which crops are adapted will require the transfer of genes from wild populations adapted to new environments or the use of novel genes. Crop species are derived for many different flowering plant groups but many are from a small number of families (e.g., Poaceae and Fabaceae). Crop plants have many traits that reflect the environments in which they evolved prior to domestication. Humans have collected plants for food for a long period of time prior to domestication of plants and the establishment of agriculture in the last 10,000 years. Pre-domestication use of plants by humans or natural variants that suit domestication (Ishii et al., 2013) may have also impacted upon some plant populations but domestication has usually resulted in significant genetic alteration of plants to suit human production in agriculture and food uses (Jin et al., 2008).

## CHOICE OF SPECIES FOR CLIMATE RESILIENT AGRICULTURE

Domesticated crop species are few in number compared to the total number of land plant species (Henry, 2010). A small number of plant species that have been adapted to wide scale production account for a large part of the energy and protein in human diets. These have become the key crops contributing to global food security. A larger number of species have been domesticated for more limited local production in specific regions. Some of these could be considered for adaptation to a wider range of environments.

Genomics tools provide new options for accelerated domestication of new species to allow adaptation of agriculture to climate change (Shapter et al., 2013).

### MONO-PHYLETIC AND POLYPHYLETIC DOMESTICATION

Domestication may have been a single genetic event with all the domesticated plants being descendent from the same wild parents or have involved a few or many independent domestication events with many wild plants contributing to the domesticated gene pool. This understanding may provide the opportunity to repeat the domestication of important crop species from a different or more diverse gene pool. Genome analysis may be used to guide this process.

### CENTERS OF ORIGIN

The center of origin of a crop species is the region from which the species is believed to have been domesticated. These are the environments that the crop plant was originally best adapted to survive at the time of domestication. Domestication from a different population selected by genome analysis may provide an opportunity to develop genotypes adapted to a new environment.

### CENTERS OF DIVERSITY

Genome analysis allows rapid identification of geographic centers of genome diversity. The center of diversity of a crop species is the region displaying the greatest genetic diversity of the crop species or its wild relatives. This may be distinct from the center of origin as plant species may have been domesticated in areas that are not those including the greatest diversity. Identification of these locations may provide new and diverse germplasm and define new environments for production of the crop now or in the future. Asian rice (*Oryza sativa*) was probably domesticated in China from wild *O. rufipogon*. The A genome clade of wild rice relatives is now considered to be most diverse further south with a center of diversity in New Guinea, Australia, and Indonesia. These locations may prove to be good sources of novel germplasm for rice improvement. Species from more temperate regions could be used to adapt rice to production in cooler climates.

### PRIMARY, SECONDARY, AND TERTIARY GENE POOLS

The gene pools of crop species may be considered at several levels. Genomic analysis may have value at all of these levels. The primary gene pool is the gene pool of the plant found in domestication and usually the species from which the crop was domesticated. The primary gene pool includes those plants that are available for direct use in genetic improvement of the species. The secondary gene pool may include more diverse material from other species that can be accessed but with a greater degree of difficulty. This often includes other species in the same or a related genus (Dillon et al., 2007). The tertiary gene pool is a wider group of plants from which genes can be accessed but only with significant difficulty (e.g., plants in the family outside the genus that can be accessed as a source of new genes but only with technological intervention). Understanding the genetic basis of domestication and the issues associated with access of genes from more difficult (or distant) relatives facilitates their use in crop improvement and in the domestication of new species to adapt agriculture to climate change (Malory et al., 2011). These analyses are more powerful at the whole genome level.

## ADVANCES IN GENOMICS OF CROPS

Advances in DNA sequencing in the last few years have resulted in genomic sequence data becoming more readily available (Edwards et al., 2012). Major efforts have been made to produce reference genome sequences for key species. This allows rapid analysis of sequence variation within species. However, *de novo* assembly of sequence data may be necessary to detect all differences and advances in sequencing technology to make this routinely possible with large plant genomes will be a significant advance.

Analysis of the genomes of plants growing along environmental gradients may provide a greater understanding of how plants adapt to climate variation under natural selection (Cronin et al., 2007; Fitzgerald et al., 2011; Shapter et al., 2012).

## GENOMIC ANALYSIS OF GENETIC RESOURCES

Analysis of the genomes of plant genetic resources will become a key tool to enable their utilization in crop improvement for climate adaptation. Targeting of genetic resources from environments that match the one being breed for is an important strategy. Large scale sequencing of accessions in plant germplasm collections will provide a platform to enable these approaches (Henry, 2013).

Increased utilization of wild crop relatives will remain a major strategy for adaptation of crops to the environmental factors associated with climate change. Many crop wild relatives remain poorly collected and are not yet represent well in seed banks. Climate change and human development risk loss of this genetic diversity making accelerated collection of crop wild relatives urgent. Rice illustrates this challenge. The closest wild relatives of rice are those from the A genome clade from which rice was domesticated (Vaughan et al., 2006). Recent research has identified two possible new species in this group that represent important new sources of diversity for rice improvement (Sotowa et al., 2013). Rice wild relatives from some regions such as Africa (Wambugu et al., 2013) and Australia (Henry et al., 2010) are poorly known.

## ANALYSIS OF NATURAL POPULATIONS AS A GUIDE TO IMPROVEMENT OF CROPS FOR AGRICULTURAL PRODUCTION

The analysis of populations of wild relatives of barley (Cronin et al., 2007; Fitzgerald et al., 2011) and rice (Fitzgerald et al., 2011; Shapter et al., 2012) indicate the potential value of genome analysis of these populations to support efforts to develop crop varieties adapted to new climates.

In these studies, wild plants were collected from diverse environments or along a sharp environmental gradient. Sampling of the same population over time as the climate changes could be simulated by this strategy. In only a few cases we can access samples that have been sampled from the same population over a significant period of time. Key findings were that adaptation to hotter or dryer environments was associated with increased diversity of biotic stress genes. Coping with abiotic stress may be confounded by overriding associated changes in the biotic environment (Fitzgerald et al., 2011).

## REMOVING THE CONSTRAINT OF END USE QUALITY ON RAPID CROP ADAPTATION TO CLIMATE

Productivity gains in crop production require elimination of constraints to utilization of more diverse germplasm. In some species the requirements of end uses are a major limitation. Market requirements for specific food or processing attributes that are complex or not well understood at the genetic level can greatly hamper attempts to use diverse adapted germplasm. Genomics tools that allow these traits to be readily selected for in breeding will assist by removing these as constraints to rapid climate adaptation (Henry, 2014). Food quality traits are often associated with human selection in domestication. They are often relatively simply controlled genetically because of their relatively recent and brief evolution under human selection in the last 10,000 years or less. Improved understanding these genes can be targeted as achievable steps toward removing a major constraint on climate adaptation.

## AVOIDING SELECTION THAT REDUCES CLIMATIC RESILIENCE

Human selection for quality may result in loss of environmental adaptation. Fragrance in rice is highly attractive to humans and adds significant value to rice. The sequencing of the rice genome allowed the identification of the genetic basis of this trait (Bradbury et al., 2005) due to the gene being flanked by closely linked known markers (Qingsheng et al., 2003). The gene responsible is an aldehyde dehydrogenase (Bradbury et al., 2008) the activity of which is lost in fragrant genotypes. The loss of the gene reduces the ability of the plant to cope with salt stress (Fitzgerald et al., 2010). Whole genome understanding of genes responsible for quality (Kharabian-Masouleh et al., 2012) will allow their relationship to abiotic stress tolerance genes to be carefully evaluated. Very attractive traits like fragrance may require strategies such as selection of compensating abiotic stress tolerance genes to counteract the deleterious effects of the quality gene.

## DURABLE PEST AND DISEASE RESISTANCE IN A CHANGING CLIMATE

The breeding of crops to cope with new pests and diseases will be a key strategy to allow plants to cope with new climates. Genes from wild populations will continue to be a major option but this may need to be complemented by the use of novel transgenes or genetic modifications.

## ROLE OF CONTINUING TECHNOLOGY ADVANCES

Technology advances will continue to be critical. Ultimately we need to be able to access whole genome information on all crop species and their wild relatives to be effective in rapid crop adaptation to climate. Ongoing developments in the chemistry of DNA sequencing and in information technology hardware and software will be required to allow these very large amounts of information to be captured and managed.

## Conflict of Interest Statement

The author declares that the research was conducted in the absence of any commercial or financial relationships that could be construed as a potential conflict of interest.
